# George Henry Temple

**Published:** 1940

**Authors:** 


### GEORGE HENRY TEMPLE, M.B., C.M.

By the death on 24th January, 1940, at the age of 75, of George Temple, Weston-super-Mare and the surrounding district have lost a much-loved and respected friend, as well as an outstanding surgeon. A bachelor, generous and kind-hearted, he was full of vitality almost to the day of his death.

George, with his wonderful dexterity and keenness of mind, was for 35 years the soul of the hospital for which he worked so unceasingly and untiringly.

Educated at Repton, he took his degree at Edinburgh University in 1888. He became House Surgeon to the Weston-super-Mare Hospital in 1895, and in 1897 set up in practice in the town and almost at once obtained the posts of Poor Law and Police Court Medical Officer. In 1905 he was appointed as Surgeon to the Hospital and remained on the active staff until his death.

He died after a few days' illness in the Hospital he had loved and served so long and faithfully.

As a younger man he was a very keen tennis player, an excellent shot, and interested in amateur theatricals, in which he took an active part. A wonderful billiards and snooker player, he held his position as one of the best players in the town almost until the time of his death.

For thirty-six years George Temple was Chief Officer of the Fire Brigade, and only relinquished the post at the outbreak of the present war, when the appointment of a whole-time officer became necessary.

At his funeral the coffin was carried on the fire-engine, and the Brigade formed a guard of honour.

He took an active interest in Freemasonry, and was a Founder and Past Master, King Alfred Lodge, No. 3169; Member of St. Kew Lodge, No. 1222; Past Provincial Junior Grand Warden (Somerset); Past Z of the Inkerman Chapter, 1222, and Past Provincial Grand Registrar of the Royal Arch Chapter of Somerset; a former Member and Past Master of the Else Mark Lodge No. 102; Past M.W.S. of the William De Irwin and of the 30th Degree of the Ancient and Accepted Right Rose Croix; P.E.P. of the Worlebury Preceptory of St. Dunstan; and P.P. Banner Bearer of the Provincial Priory of Somerset, Monmouth and South Wales—Knights Templars.

He became a member of the B.M.A. in 1888 and was Chairman of the East Somerset Division in 1933 and 1934, and a member of the present Local Medical War Committee.

Many a young practitioner has benefited by his kindly criticism and advice.

He will long be mourned and missed by the many he helped, directly and indirectly.

